# Tire-Pressure Identification Using Intelligent Tire with Three-Axis Accelerometer

**DOI:** 10.3390/s19112560

**Published:** 2019-06-05

**Authors:** Bing Zhu, Jiayi Han, Jian Zhao

**Affiliations:** State Key Laboratory of Automotive Simulation and Control, Jilin University, Changchun 130025, China; zhubing@jlu.edu.cn (B.Z.); jyhan18@mails.jlu.edu.cn (J.H.)

**Keywords:** intelligent tires, tire pressure, accelerometer, wheel vibration

## Abstract

An intelligent tire uses sensors to dynamically acquire or monitor its state. It plays a critical role in safety and maneuverability. Tire pressure is one of the most important status parameters of a tire; it influences vehicle performance in several important ways. In this paper, we propose a tire-pressure identification scheme using an intelligent tire with 3-axis accelerometers. As the primary sensing system, the accelerometers can continuously and accurately detect tire pressure with less electronic equipment mounted in the tire. To identify tire pressure in real time during routine driving, we first developed a prototype for the intelligent tire with three 3-axis accelerometers, and carried out data-acquisition tests under different tire pressures. Then we filtered the data and concentrated on the vibration acceleration of the rim in the circumferential direction. After analysis, we established the relationship between tire pressure and characteristic frequency of the rim. Finally, we verified our identification scheme with actual vehicle data at different tire pressures. The results confirm that the identified tire pressure is very close to the actual value.

## 1. Introduction

Interactions between a tire and the road exert forces on a vehicle and alter its motion state. The ultimate aim of most vehicle active safety systems, such as the anti-lock braking system (ABS) or the electronic stability program (ESP), is to make the tires controllable [1,2,3]. The status information of the tire is especially important for all kinds of active safety systems and especially for self-driving vehicles [4,5].

At present, tire information, such as tire force and coefficient of road adhesion, is estimated by modules and filters [6,7,8,9,10]. During estimation, there is much simplification and linearization in the models of the vehicle, wheel, and tires, and the kinematic state of the vehicle is indeterminate. Therefore, application of estimated tire information is always restricted.

To acquire more accurate and appropriate tire information, the concept of an intelligent tire system is being researched extensively [11,12]. The intelligent tire, which is equipped with sensors and other electronic equipment, is meant to measure the tire pressure, deformation, force, and other information. Its most important part is the sensing system [13,14]. Intelligent tires can be classified into five major types, based on their sensing methods, which are accelerometers, optical sensors, strain sensors, polyvinylidene fluoride (PVDF) sensors, and other sensors.

Yi Xiong et al. used an optical tire sensor-based laser to measure rolling tire deformation [15]. Matsuzaki et al. calculated the in-plane strain and out-of-plane displacement with a single CCD camera fixed on the wheel rim [16]. Optical sensors can achieve noncontact measurement, hence they have no adverse effect on the tire rubber, but these sensors are expensive and consume too much power, and wheel vibration can influence their accuracy.

Strain sensors and PVDF sensors have gained more attention in tire sensing [17,18,19,20,21]. Zhang et al. embedded a pressure-sensitive, electric conductive rubber sensor inside the tire rubber layer to extract the 3-D forces on the contact patch [17]. Armstrong et al. applied three piezoelectric film sensors, which are a kind of strain sensor, on the inner surface of a tire to detect normal pressure, deflection, and longitudinal strain [18]. Erdogan et al. proposed a method to estimate the tire-road friction coefficient using a sensor consisting of PVDF film attached to the surface of a cantilever beam [19]. However, these sensors occupy a large area of the inner surface of the tire, which will alter the elastic property of the rubber and cause abnormal measurements. In addition, the huge temperature variation of a tire has a strong impact on strain sensors and PVDF sensors.

Accelerometers are now used widely for intelligent tires [22,23,24,25]. Savaresi et al. presented a direct approach to estimate the instantaneous vertical and longitudinal forces by in-tire acceleration measurements and standard wheel-speed sensors [22]. Matsuzaki et al. proposed an identification scheme for the friction coefficient involving the slip angle, and applied force estimation through the use of a 3-axis accelerometer glued on the inner surface of a tire [23]. Niskanen et al. studied the distorted contact area of a tire in wet conditions using three accelerometers fixed on its inner liner [24]. Accelerometers are compact, small, and weigh just a few grams. They hardly influence a tire when glued to its inner surface. Accelerometers also consume less energy than optical or other sensors. This makes it possible to achieve a passive intelligent tire. In addition, their output signals are easy to read and contain more information compared to PVDF or strain sensors. With all of these attractive features, accelerometers are the most suitable sensors for intelligent tires.

Intelligent tires equipped with accelerometers, as described above, are mostly researched to identify and estimate tire deformation, slip angle, vertical or longitudinal force, and tire-road friction coefficients. While this information is vital, there has been little research on tire pressure using intelligent tires equipped with accelerometers. Tire pressure influences several important aspects of vehicle performance [26]. Rolling resistance increases when a tire loses pressure, which reduces fuel economy. Pressure that is too low results in stationary wear, which can cause a tire to burst. When the tire pressure is high, the tire and road contact patch will be non-uniform, which reduces adhesive force. In addition, the tire will harden, which leads to an uncomfortable ride for passengers. The tire pressure also makes sense to vehicle active safety or control applications. The cornering stiffness is influenced by the tire pressure greatly. The cornering stiffness of the front and rear tires determines the steering behavior directly [27,28]. In this consideration, the tire pressure has something to do with the active safety systems, such as ESP.

The tire pressure monitoring in real time becomes more and more important for the present and the future. The vehicle control algorithm is more and more complexity, and more variables are taken into account during tire modelling. The influence of the tire pressure can be and must be taken into consideration. For example, Janulevicius et.al. investigated how driving wheels of a front-loaded vehicle interact with the terrain depending on tire pressure [29]. Toma et al. studied the influence of tire pressure on the results of diagnosing brakes and suspension [30]. With the real time tire pressure, the control strategy under different tire pressure can be optimized and enhances the vehicle performance [31]. Therefore, Tire-pressure monitoring should be the fundamental function of an intelligent tire. Tire-pressure monitor systems (TPMSs) are widely used all over the world [32,33].

There are two types of TPMS. One is called indirect TPMS and the other is direct TPMS. The indirect TPMS can work with no need for installing additional sensors. It uses the signal of the wheel speed sensors or other existing sensors [34]. This kind of TPMS is only able to judge whether the tire is lacking of pressure. It cannot provide the precise tire pressure in real time. Therefore, the use of the indirect TPMS is limited. It can only help to acquaint the drivers with the tire situation and it can do nothing with the advanced active safety systems [35]. The direct TPMS is based on a pressure sensor mounted at the tire nozzle. It is practical and durable for the present, but it is a separate system. More electronic circuits or equipment are acquired to combine this kind of TPMS with intelligent tire or advanced active safety systems. This will reduce the reliability and add complexity. If we can estimate the tire pressure precisely in real time using the sensors installed in the intelligent tire, a seamless tire pressure monitoring function will be achieved. In the future when the intelligent tire with multi-function is developed by equipping accelerometer or other vibration sensors, the tire pressure identification method proposed in this paper will help to take the place of the present TPMS. The major motivation to install accelerometers in the tire may be to estimate tire deformation, slip angle or frictional force. In this condition, if we can identify the tire pressure by the accelerometers which is already installed in the tire, then pressure sensors in the tire will be unnecessary. The less electronic equipment we mount in the tire, the more practical and reliable an intelligent tire system we can achieve [5,36].

In this paper, we have tried to identify the tire pressure using 3-axis accelerometers in the tire. We designed a development platform for an intelligent tire, and conducted road tests under different tire pressures for data acquisition using the platform. We mathematically processed the accelerometer data and developed an identification scheme for tire pressure by analyzing the characteristics in the frequency domain. To verify our tire-pressure identification scheme, we conducted another road test was carried out with different tire pressures and compared the identified and actual tire pressures.

## 2. Intelligent Tire with 3-Axis Accelerometers and Data-Acquisition Test

### 2.1. Test Equipment and Method

**Figure 1:**
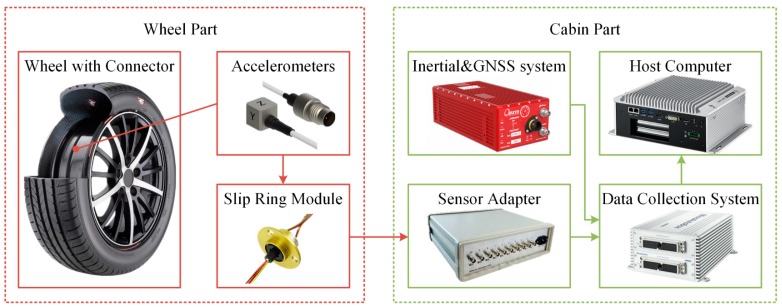
Overall structure of intelligent tire.

To discover the pattern of wheel- and tire-vibration acceleration, we made a prototype for an intelligent tire with 3-axis accelerometers; the overall structure is shown in Figure 1. The wheel was 16 × 6.5 J and the tire was a 205/55R16 (Michelin, Clermont-Ferrand, France). The wheel part contained a wheel with connector and accelerometers, and a slip ring module to conduct an electrical signal. The cabin part contained a sensor adapter, data-acquisition system, host computer, and inertial system and global navigation satellite system (GNSS).

**Figure 2:**
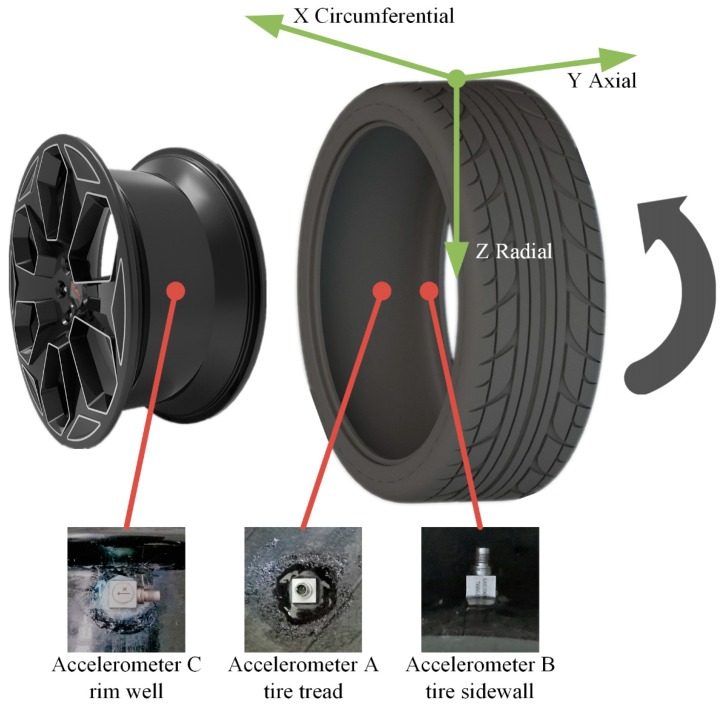
Accelerometer setup and tire coordinates.

We used three 3-axis accelerometers to measure the vibration acceleration of different parts. The 3-axis accelerometers (SAE30050Q, Yutian, Wuxi, China) were small and light (9.5 mm × 9.5 mm × 9.5 mm, 6 g), with measurement ranges ±500 g and 2~5000 Hz. Accelerometer C was fixed to the well of the rim, and accelerometers A and B were respectively fixed to the internal surface of the tread and the sidewall of the tire. The setup of the accelerometers and the coordinates of the wheel are shown in Figure 2.

**Figure 3:**
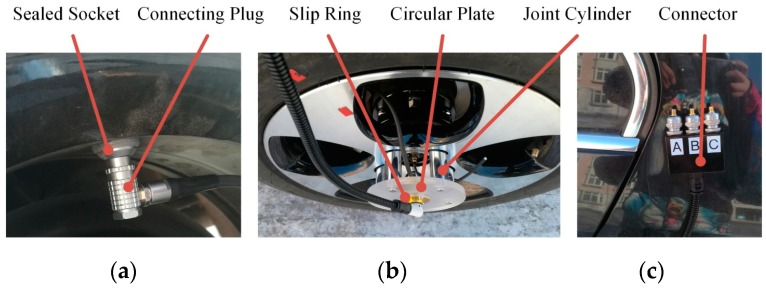

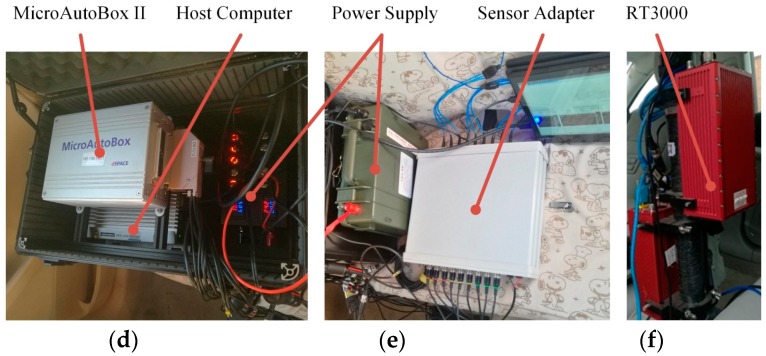
Test setup and equipment: (**a**) Connector on the rim well; (**b**) slip ring module; (**c**) connector on the vehicle body; (**d**) data-collection system; (**e**) battery and adapter; (**f**) combined inertial and global navigation satellite system (GNSS) system.

To export the signal from inside the tire, we drilled on the well and installed a sealed connection socket, as shown in Figure 3a. The maximum sealing pressure of the socket was 60 bars, which exceeded the maximum tire pressure of 3.5 bars. The socket introduced no possibility of leaks from the tire and transmitted electrical signals without loss.

Because a wheel constantly spins during driving, we designed a slip ring module to transmit the signal from the wheel to the car body, as shown in Figure 3b. The slip ring was assembled in a circular plate, which was attached to the wheel hub by five joint cylinders, each with a claw at one end to clamp the nut of the hub bolt and a threaded hole at the other end to attach the circular plate.

We used integral electronic piezoelectric (IEPE) accelerometers, so a sensor adapter (CT5210, CHENGTEC, Shanghai, China) was needed to supply power and a condition signal, as shown in Figure 3e. 

An RT3000 (OxTS, Middleton Stoney, United Kingdom) combined inertial and GNSS system was installed in the vehicle, as shown in Figure 3f, to acquire vehicle dynamic states, such as velocity, acceleration, and yaw rate. The velocity accuracy was 0.05 km/h, and the data frequency was 100 Hz.

We used a MicroAutoBox II (dSPACE, Paderborn, Germany) data-acquisition system to record the vibration acceleration, vehicle speed, and other data, as shown in Figure 3d. This device has 32 16-bit analog input channels with a 200 Ksps conversion rate and −10~10V input voltage range, eight 16-bit analog output channels, and six controller area network (CAN) channels. The outputs of the accelerometers were conditioned by the adapter and then input to the MicroAutoBox II through an analog-to-digital converter. The RT3000 and MicroAutoBox II communicated by CAN (Controller Area Network) bus. The data were acquired through a CAN channel.

### 2.2. Vehicle Test under Different Tire Pressures

To determine how to identify the tire pressure in an intelligent tire, we conducted an experiment in which the test vehicle traveled on an urban road with different tire pressures. The tire pressure was set at 150 kPa, 250 kPa, and 350 kPa. To include a variety of road surfaces and to develop a practical and universal method for tire pressure identification, we chose a road of about 2 km, including asphalt, cement, damaged, and snow-covered road surfaces, and the test vehicle traveled at speeds from 0 to 50 km/h in a natural fashion at each tire pressure. The weather was sunny and the temperature was about −20 °C. 

**Figure 4:**
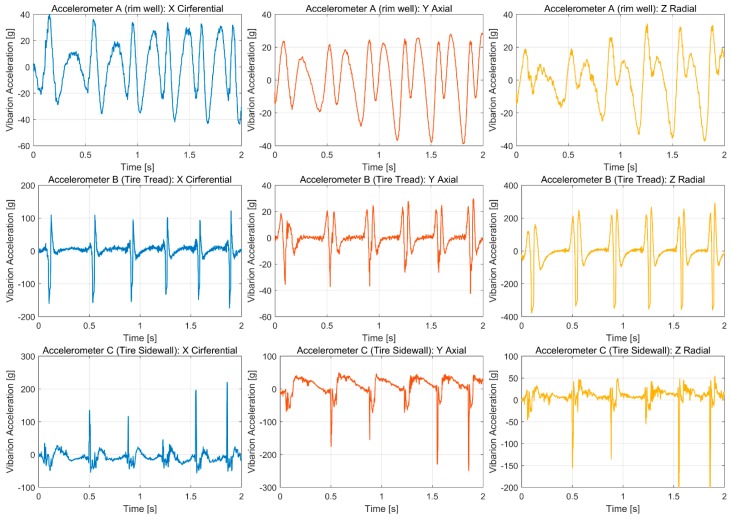
Vibration acceleration of rim well, tire tread, and sidewall in three directions.

A truncated section of the resultant data is shown in Figure 4. The data include nine channels of vibration acceleration from three 3-axis accelerometers, and the velocity, acceleration, yaw rate, and other parameters were acquired synchronously.

## 3. Identification Scheme for Tire Pressure

The wheel, which is the combination of the rim and tire, is a kind of spring-damp system. That means a change of stiffness will alter the characteristic frequency of the wheel. According to the spring-loaded ring tire model in the vibration model [37,38], the relationship between torsion vibration natural frequency and tire stiffness can be derived as:

(1) $$f_{tor}=\frac{1}{2\pi }\sqrt{\frac{K_{\theta }}{\rho bl}},$$

where *f_tor_* is the torsion natural frequency, which can reflect the characteristics of the circumferential vibration; *K_θ_* is the rotational spring constant of the sidewall portion per unit length, representing the tire and wheel stiffness; *ρ* is the density; and *b* and *l* are the thickness and half-length, respectively, of the tread ring.

From Equation (1), we can see that the natural frequency increases with the stiffness. Furthermore, the stiffness of the wheel can be influenced by the tire pressure. Another tire model from Akasaka illustrates the relationship between stiffness and tire pressure [39]. The theoretical formula of the rotational spring constant about p is [38]:

(2) $$K_{t}=g\left(r_{B},r_{c},r_{D},\varphi_{D},\alpha_{D},\theta \right)\cdot p+h\left(h,r_{B},r_{D},\varphi_{D},V_{r},G_{r}\right),$$

where *K_t_* is the rotational spring constant, which is nearly the same as *K_θ_* in the spring-loaded ring tire mode; *p* is the tire pressure; *r_B_*, *r_C_*, and *r_D_* are the tire radius parameters related to special tire positions; *φ_D_*, and *α_D_* are the angle parameters; *θ* is the rotational displacement; *V_r_* is content rate; *G_r_* is the rigidity modulus; and *h* is the thickness. Equation (2) is used to illustrate the relationship between the stiffness and tire pressure, so there is no need to derive it in detail.

Generally, the stiffness of the wheel increases with the tire pressure and when the tire pressure continues to increase, the stiffness will remain unchanged or will decrease slightly.

According to this principle, we can identify the tire pressure by mining the data provided by the intelligent tire, and we can determine the characteristic frequency. Above all, it is important to determine how the tire pressure influences the stiffness and establish an accurate relationship between characteristic frequency and tire pressure.

### 3.1. Vibration Analysis of the Tire

Previous research has shown that one of the characteristic frequencies of the wheel is between 35 Hz and 80 Hz [40,41]. Therefore, we concentrated on analyzing the amplitude-frequency characteristic in this range.

We first analyzed the vibration acceleration data under different tire pressures from accelerometers A and B. A piece of data under a steady speed was selected to show the frequency component of the tire vibration.

We used the fast Fourier transform (FFT) algorithm to achieve a time-frequency transformation. FFT is an improved algorithm for the discrete Fourier transform (DFT). The main formulas are as follows:

(3) $$\begin{array}{c} X\left(k\right)=X_{1}\left(k\right)+W_{N}^{k}X_{2}\left(k\right)\\ X\left(k+\frac{N}{2}\right)=X_{1}\left(k\right)-W_{N}^{k}X_{2}\left(k\right) \end{array},$$

where:

(4) $$\begin{array}{c} X_{1}\left(k\right)=\overset{N/2-1}{\underset{r=0}{\sum }}x_{1}\left(r\right)W_{N/2}^{kr}\\ X_{2}\left(k\right)=\overset{N/2-1}{\underset{r=0}{\sum }}x_{2}\left(r\right)W_{N/2}^{kr} \end{array},$$

where:

(5) $$W_{N}^{kn}=e^{-j\frac{2\pi }{N}kn},$$

where *W* is the twiddle factor, *x_1_*(*r*) and *x_2_*(*r*) are subsequences of the time series data dividing by the odevity of the ordinal number. *X*(*k*) and *X*(*k + N/2*) are the frequency domain results.

**Figure 5:**
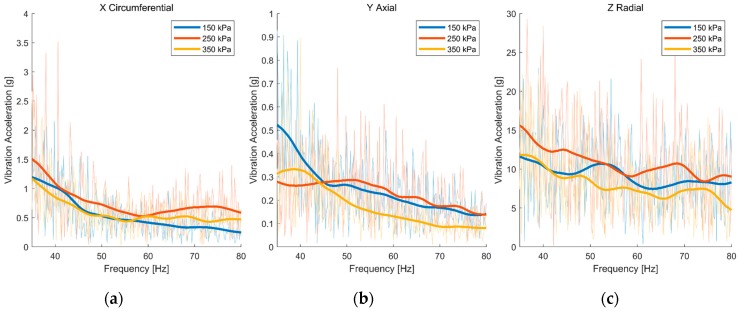
Amplitude-frequency curve of accelerometer A (tire tread) in three directions: (**a**) Circumferential direction; (**b**) axial direction; (**c**) radial direction.

**Figure 6:**
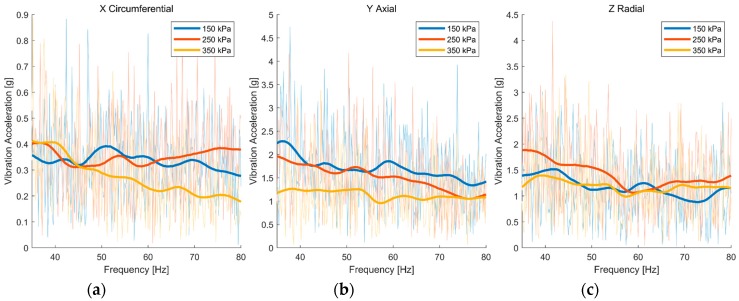
Amplitude-frequency curve of accelerometer B (tire sidewall) in three directions: (**a**) Circumferential direction; (**b**) axial direction; (**c**) radial direction.

The length of the data was 5 seconds, and the sample frequency was 1 kHz. The results are shown in Figure 5 and Figure 6. The semitransparent spectrums are the original results of the FFT, and the weighted curves are the smoothed results by Gaussian smoothing filter. The vibrations of the tire tread were almost alike in three directions under different tire pressures, and the tire side wall was the same. It is difficult to distinguish between tire pressures through spectral analysis. We speculated that the tire had filtered the vibration through its elastic property. The changes in tire pressure did not lead to the obvious differences of the vibration characteristic of the tire.

### 3.2. Vibration Analysis of the Rim

**Figure 7:**
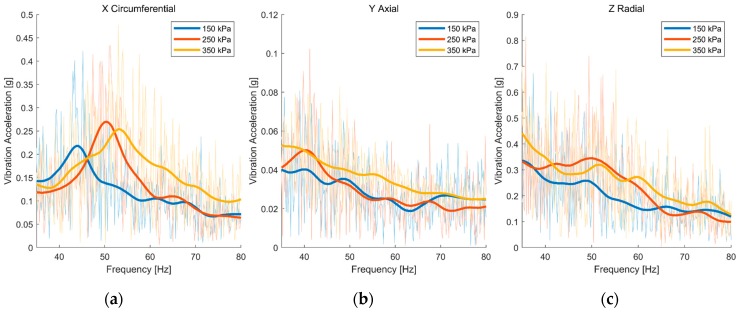
Amplitude-frequency curve of accelerometer C (rim well) in three directions: (**a**) Circumferential direction; (**b**) axial direction; (**c**) radial direction.

We analyzed the data from accelerometer C, which reflected the vibration of the rim. As a rigid body, the rim will vibrate with a regular pattern when excited by the road surface. The data of accelerometer A were processed in the same way. The result is shown in Figure 7. It is observed that the axial and radial vibration amplitude-frequency curves remain the same for accelerometers A and B, reflecting no distinction. However, the result in the circumferential direction reveals a difference in the peak frequency at different tire pressures. The peak frequency rises with the tire pressure. The changes in tire pressure are reflected in the peak frequency of the amplitude- frequency curve. Therefore, the circumferential vibration of the rim becomes the key for tire-pressure identification. To prove that the change of the peak frequency is not occasional, we analyzed the relationship between peak frequency and tire pressure over the whole vehicle test.

### 3.3. Relationship Between Characteristic Frequency and Tire Pressure

**Figure 8:**
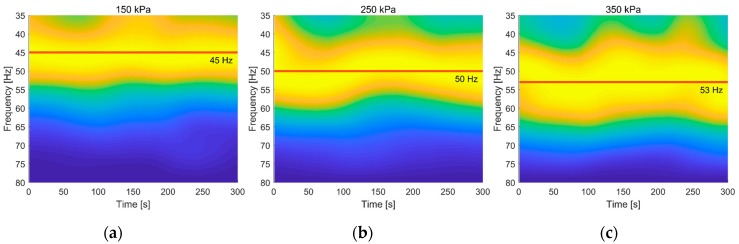
Segmented amplitude-frequency curve of accelerometer A (rim well) in circumferential direction: (**a**) 150 kPa; (**b**) 250 kPa; (**c**) 350 kPa.

The circumferential acceleration data of accelerometer C under tire pressures of 150 kPa, 250 kPa, and 350 kPa were segmented every five seconds, and each segment was processed through FFT. We truncated the amplitude-frequency curves from 35 Hz to 80 Hz, then smoothed and normalized each curve and plotted them in chronological order. The result is shown in Figure 8. The vibration acceleration intensity at different frequency is represented by colors. The intensity increases from blue to yellow. The red weighted straight lines represent the average peak positions at different tire pressures.

Figure 8 shows the peak values around 45 Hz at a tire pressure of 150 kPa. As the tire pressure increased, the peak values went to about 50 Hz when the tire pressure was 250 kPa. When the tire pressure increased to 350 kPa, the peak values gathered around 53 Hz. This trend is consistent with the analysis presented at the beginning of this section. That is to say, the characteristic frequency about which the peak values gathered is an inherent attribute of the wheel. It will not be affected by external factors such as vehicle speed, but increases with tire pressure within a certain range. Therefore, we next sought to establish an accurate rule or relationship between characteristic frequency and tire pressure.

**Figure 9:**
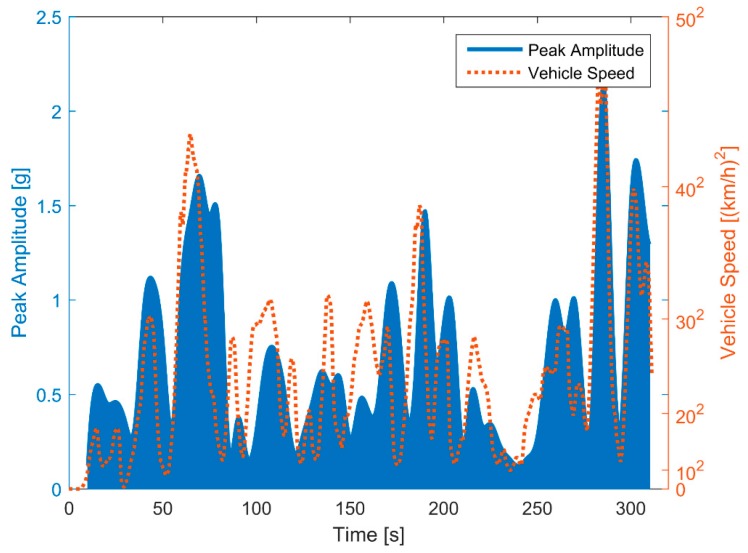
Relationship between peak amplitude and vehicle speed.

To determine the characteristic frequency at different tire pressures, it is practical and effective to combine all segments of amplitude-frequency curves and locate the peak value. However, we first must consider how the vehicle speed affects the amplitude of the amplitude-frequency curves, or the features of the amplitude-frequency curves at low vehicle speed will be neglected. Because the vibration acceleration intensity is influenced greatly by the speed. The wheel and tire will be excited more greatly when the speed goes higher [42]. We will use the segmented amplitude-frequency data at 350 kPa to illustrate the effect of vehicle speed on peak amplitude. We found that the peak amplitude is directly proportional to the square of the vehicle speed, as shown in Figure 9.

According to this relationship, we developed a function to calculate the weighted amplitude-frequency curves at different tire pressures:

(6) $$G_{sum,tp}=\sum^{}\frac{1}{v_{i}^{2}}G_{tp,i},$$

where *G_sum,tp_* is the weighted amplitude-frequency curve (*tp* = 150 kPa, 250 kPa, 350 kPa); *G_tp,i_* is the segmented amplitude-frequency curve (*i* = 1,2,3,…); *1/v_i_^2^* is the weight function to eliminate the effect of vehicle speed; and *v_i_* is the average vehicle speed during the current segment.

**Figure 10:**
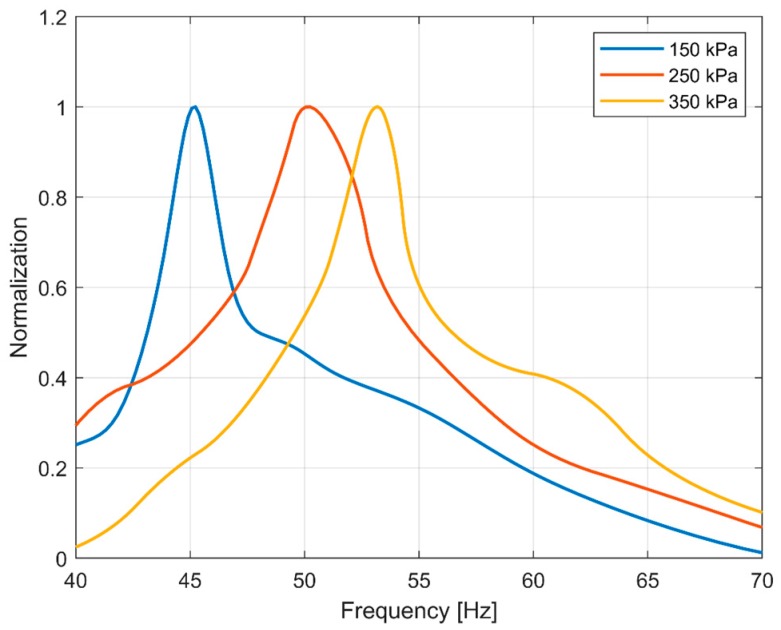
Weighted amplitude-frequency curves at different tire pressures.

The weighted amplitude-frequency curves at different tire pressures were smoothed and normalized for convenient observation. The results are shown in Figure 10. The characteristic frequencies at 150 kPa, 250 kPa, and 350 kPa are easily observed to be 45.4 Hz, 50.2 Hz, and 53.2 Hz, respectively.

**Figure 11:**
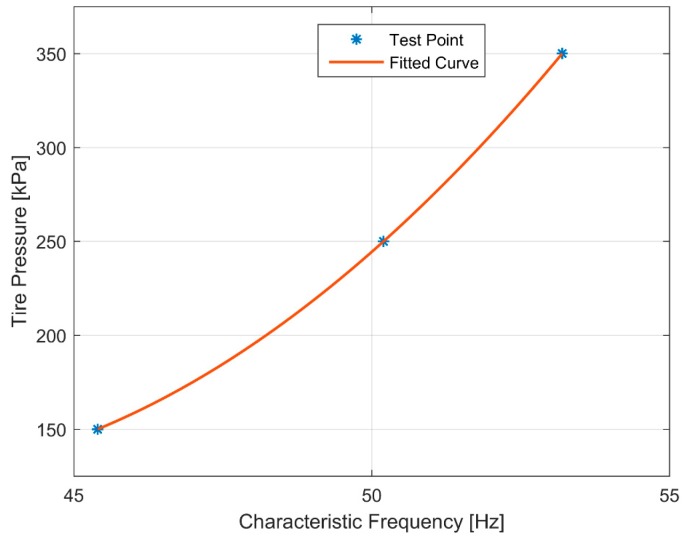
Relationship between characteristic frequency and tire pressure.

We used frequency as the independent variable and tire pressure as the dependent variable, and used the least square method to fit the relationship between characteristic frequency and tire pressure. The curve is shown in Figure 11, and the relation function is shown as Equation (7).

(7) $$p_{tire}=1.60\cdot f_{peak}^{2}-132.37\cdot f_{peak}+2856.54,$$

where *p_tire_* is the identified tire pressure and *f_peak_* is the characteristic frequency.

## 4. Verification and Discussion

To verify the scheme developed in Section 3, we conducted another experiment, which was the same as the experiment in Section 2, except that the tire pressures were set at 200 kPa and 300 kPa.

**Figure 12:**
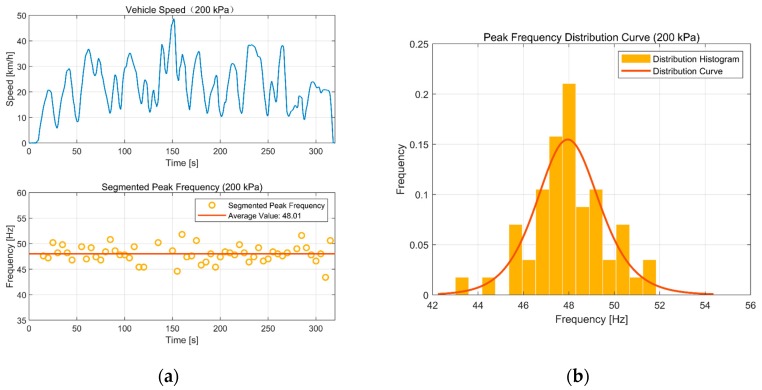
Result at 200 kPa: (**a**) Peak-frequency extraction; (**b**) peak frequency distribution curve.

We first broke the circumferential vibration acceleration data into small segments of five-seconds duration. We transformed each segment from the time to frequency domain by FFT, smoothed the amplitude-frequency curve, and extracted the peak frequency. Figure 12 shows the result at 200 kPa.

We can infer that there is no relation between peak frequency and vehicle speed. Whatever speed the vehicle travels, the peak frequency is steady and easily determined. This is consistent with the analysis in Section 3. The probability of an identification result whose error is below 5% compared with the actual tire pressure 200 kPa is 85%. The average value of the peak frequency during the experiments was 48.01 Hz. Using the result of Section 3, the identified tire pressure is determined as 195.39 kPa through Equation (4). The identification error is 2.31%.

There is no denying that the instantaneous peak-frequency extraction is not accurate enough, so we analyzed the errors, as shown in Figure 12b. The errors are normally distributed. So, we can enhance the identification precision by averaging several continuous peak frequencies.

**Figure 13:**
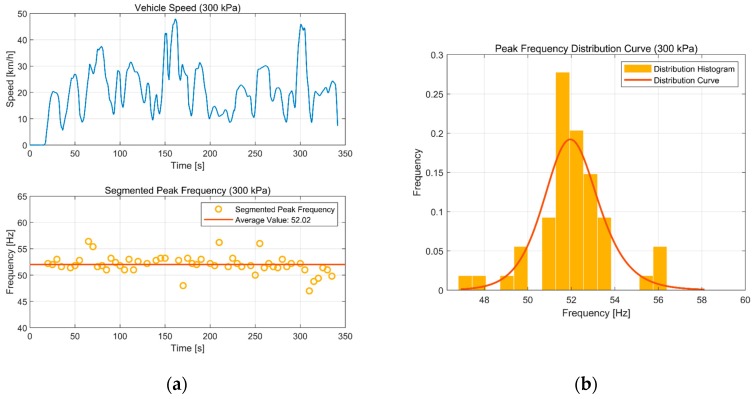
Result at 300 kPa: (**a**) Peak-frequency extraction; (**b**) peak frequency distribution curve.

We did the same with the data at 300 kPa. Figure 13a shows the identification of the peak frequency. The probability of an identification result whose error is below 5% compared with the actual tire pressure of 300 kPa is 83%. The average peak frequency was 52.02 Hz, and the identified tire pressure is computed as 300.38 kPa through Equation (4). The identification error is 0.13%. Figure 13b shows the error distribution of the peak-frequency identification. It is much the same as the result at 200 kPa.

These results prove that the tire pressure identification scheme using vibration acceleration we developed is able to estimate the tire pressure accurately. The specific value of the tire pressure can be given in real time using this method, rather than a simple tire pressure level.

## 5. Conclusions

To “intelligentize” a tire, which means that the tire itself is capable of sensing and communicating, we designed a prototype of an intelligent tire with three 3-axis accelerometers. To develop a tire-pressure identification scheme, we conducted a data-acquisition experiment using the prototype at different tire pressures and analyzing the wheel vibration in the frequency domain. We discovered a relationship between tire pressure and tire vibration. Generally, the peak frequency rises with the tire pressure. The identification scheme was verified with actual vehicle data at different tire pressures. The results confirm that the identified tire pressure is very close to the actual value. The probability of identification error below 5% is above 80%. The processing complexity and period are reasonable and practical.
